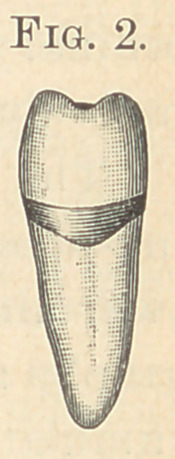# A New Method of Attaching Artificial Crowns to Badly Decayed Roots

**Published:** 1895-09

**Authors:** George H. Chance

**Affiliations:** Portland, Oregon


### A NEW METHOD OF ATTACHING ARTIFICIAL CROWNS TO BADLY DECAYED ROOTS.[1]

BY GEORGE H. CHANCE, D.D.S., PORTLAND, OREGON.

In these days of progressive dental surgery, when so much is being accomplished along conservative lines, and when, in addition to other work, very many badly decayed and, to the superficial observer, apparently useless roots of teeth are being restored to health and usefulness, it does not seem strange that the patrons of the dental chair should, as the years roll on, become more and more exacting in their demands upon the skill of the dental surgeon; neither is it strange that when one is listening to such appeals as "Do save that root and put a crown on it!" he should, in response to such an appeal, attempt feats which, to the less skilful and less conservative practitioner, would probably be relegated to the domain of the impossible. This is very apt to be true in cases where the natural crown is entirely gone, and the root so badly broken down and decayed as to be completely covered up by the overgrowing gum tissue. Nevertheless, a varied experience in this special line of practice has led me to believe that, when it is desirable, almost any badly decayed root may be saved and crowned, provided there still remains enough sound dentine to securely hold one end of a small screw-post in place,—presuming, of course, that the peridental surroundings are, or can be, restored to health; but, like all other somewhat complicated operations in the mouth, "hap-hazard" work will not do in such cases. The ground to be

---

[1] Read before the Stomatological Club of San Francisco, Cal.

traversed must be carefully and intelligently studied, and each step towards the wished-for goal must be taken with due regard to the final outcome.

With these few thoughts by way of introduction, permit me to hold your attention for a few moments while I describe a method whereby such badly decayed roots as have been spoken of may be saved and successfully crowned.

To do this in the most effectual way, I deem it best to take a typical case from my own practice and proceed to describe the operation in full.

The case referred to was that of a gentleman of middle age, and the root to be treated and crowned was a right anterior bicuspid of the upper jaw. The case, when first seen, presented the following conditions: crown entirely gone, decay extending above and beyond the ordinary gingival margin when in a normal condition, gum badly inflamed and entirely covering up the root. The first step in the operation was the removal of the excess of gum-tissue, and, ascertaining that the root still possessed sufficient strength to support a crown, the decay was removed, and the concave depression in the end of the root made smooth with round-ended engine burs, the pulp-canals in the root opened and treated in the usual manner, and the space between the entrance to the canals and the gum margins closed with cotton and Sandarach varnish. The patient was then dismissed for the time being.

At the next sitting the pulp canals were filled, and an impression of the parts was taken in modelling compound, from which a plaster cast was made, and from it dies of Melotte's metal, for the purpose of striking up a thin gold cap to fit the concavity in the end of the root, using enough gold to allow the edges of the cap to extend to the gum margin. When the cap was ready it was adjusted to the root, and, with cap in position, a second impression with "the bite" was obtained, the cap coming away with the impression, which was transferred to the plaster cast. A Bonwill crown of the proper form and shade was then selected, ground, and the upper margins bevelled so that they would just enter and be enclosed by the edges of the gold cap, giving it somewhat the appearance of a banded Richmond crown. The next step was to insert a small screw-post of the proper length in each pulp-canal, which being done, a slot was cut in the top of the cap, large enough to allow the ends of the posts to pass through. The parts were then dried, and the convex surface of the cap painted with a little thick chloro-percha and slipped over the posts to its position on the root, gentle pressure being used to force out the excess of chloro-percha, while a warm blast was thrown on to evaporate the chloroform. The crown was then cemented to its place with oxyphosphate, and the occluding end in the cavity of the crown capped with gold, the whole forming a firm and, to both operator and patient, a very satisfactory piece of work, which, after several months' use, is still doing good service.

This leads me to say that, in my opinion, the Bonwill crown is not appreciated at its true value, being satisfied that in a large number of special cases much better results can be obtained by the use of a Bonwill crown, or one constructed on the Bonwill principle, in connection with a screw-post and swaged cap to cover the end of the root, than in any other crown in which the pin is directly attached to the porcelain, as in the Logan or other similar crown; and in the event of future accident, causing fracture of the porcelain, the Bonwill crown can be easily replaced without disturbance to the root, or having to do the first work over again, as is frequently the case with pin-crowns.

Fig. 1.

Fig. 2.

I herewith submit two very short roots roughly crowned in the manner described, which will further illustrate the principle and the method of application, as well as suggest to the "bridge-worker" how such roots may be utilized in sustaining small pieces of bridge-work (Figs. 1 and 2).

## Figures and Tables

**Fig. 1. f1:**
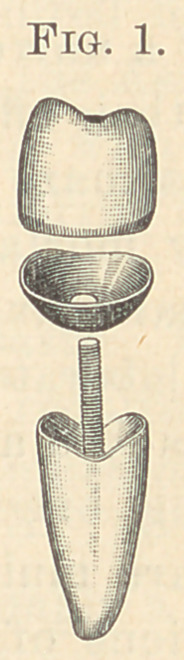


**Fig. 2. f2:**